# Epidemiology and resistance mechanisms to imipenem in *Klebsiella pneumoniae*: A multicenter study

**DOI:** 10.3892/mmr.2012.1155

**Published:** 2012-10-25

**Authors:** HUIHONG CHEN, XUE YUEHUA, WEIWEI SHEN, HUA ZHOU, LIZHONG ZHOU, ZHAOYUN LI

**Affiliations:** 1 Department of Clinical Laboratory, Central Hospital of Taizhou City, Taizhou; 2 Department of Brain Surgery, Central Hospital of Taizhou City, Taizhou; 3 Disease Control Center of Taizhou City, Taizhou; 4 Department of Infectious Diseases, First Affiliated Hospital of Zhejiang University, Hangzhou, Zhejiang; 5 Clinical Laboratory, Shanghai Ruijin Hospital, Shanghai, P.R. China

**Keywords:** gel electrophoresis in pulsed-field, *Klebsiella pneumoniae* carbapenemases, *Klebsiella pneumoniae*

## Abstract

Four clinical isolates of imipenem-resistant *Klebsiella pneumoniae* were isolated from clinical patient specimens and from samples obtained from hygienic surveillance in our hospital. We examined their minimum inhibitory concentration (MIC) to various types of antibiotics, detected the carbapenemases by a modified Hodge test and analyzed the genotype and homogeneity. The enzyme, *Klebsiella pneumoniae* carbapenemase (KPC)-2, was detected in all four isolates and this was the main cause of their imipenem resistance. In addition, these four isolates also contained the extended-spectrum β-lactamase (ESBL) gene bla_CTX-M-9_ and the cephalosporinase (AmpC) gene bla_DHA-1_, which resulted in multidrug resistance.

## Introduction

Yigit *et al* first reported *Klebsiella pneumoniae* strains with a medium to high degree of resistance to imipenem and meropenem in 2001 (1). The authors isolated a new carbapenemase which they named *Klebsiella pneumoniae* carbapenemase (KPC)-1. From then on, KPC-producing bacteria have been reported successively in various countries and regions and are now spreading globally (2–5).

An imipenem-resistant strain of *Klebsiella pneumoniae* was identified in our hospital for the first time by the ICU department on 5th July, 2008. It was detected in the blood of a patient who was transferred to our hospital following prolonged therapy in another hospital in Hangzhou. Investigations were immediately carried out to determine the source and path of the infection, and appropriate measures were taken to get the infection under control in a timely and effective manner. The aim of the present study was to analyze the resistance mechanisms and epidemiology of the imipenem-resistant *Klebsiella pneumoniae* strains isolated from the patients and hygienic surveillance samples obtained at the hospital.

Materials and methods

### Strains

Bacteriological specimens were collected from the ICU environment, medical items, hands of the medical staff and sputum and blood specimens of other patients in the ward at the Central Hospital of Taizhou City. Among the 57 collected specimens, four were identified to carry imipenem-resistant *Klebsiella pneumoniae*. The four imipenem-resistant *Klebsiella pneumoniae* clinical isolates 2011, 2163, 2193 and 2285 were investigated in this study. One was isolated from a patient’s blood specimen, two were from sputum specimens and one was from an inspection specimen which was collected from a mop used in the ward.

### Apparatus and reagents

The automatic bacteria calibrator VITEK-60 was acquired from bioMérieux SA (Marcy l’Etoile, France). Pulsed-field gel electrophoresis (PFGE) typing was performed using the CHEF Mapper^®^ XA system (Bio-Rad Laboratories, Hercules, CA, USA). Gel images were captured using a Bio-Rad GelDoc 2000 gel documentation system (Bio-Rad Laboratories). PCR was performed using a Bio-Rad PTC-200 thermal cycler (Bio-Rad Laboratories). Primers were purchased from Yingwei Jieji (Shanghai, China). Restriction endonuclease *Xba*I was purchased from Dalian Baosheng Biological Engineering Co., Ltd. (Dalian, China). SeaKem Gold Agarose was acquired from Cambrex Bioscience Rockland, Inc. (Rockland, ME, USA). Protease K was from Merck & Co., Inc. (Whitehouse Station, NJ, USA). The standard control strain H9812, susceptibility paper, meropenem, cefoperazone/sulperazon and polymyxin B were all from Oxoid (Basingstoke, UK).

### Drug susceptibility test

The minimum inhibitory concentration (MIC) was determined using a VITEK-60 automatic bacteria calibrator by the microplate dilution method. Fifteen antimicrobial agents were examined as follows: imipenem, ertapenem, cefoperazone/sulbactam, cefepime, ciprofloxacin, tobramycin, gentamicin, aztreonam, ceftazidime, ceftriaxone, piperacillin/tazobactam, cefazolin, ampicillin, ampicillin/sulbactam and cefoxitin. The agar disc diffusion method (KB method) was also used to evaluate the susceptibility of the bacteria to cefoperazone/sulperazon, polymyxin B and meropenem.

### Modified Hodge test for detection of carbapenemases

An overnight culture suspension of *Escherichia coli* ATCC 25922 adjusted to 0.5 McFarland standard was inoculated on the surface of a Mueller-Hinton agar (MHA) plate. A paper disk impregnated with 10 μg imipenem was placed at the center of the plate. The test strain was streaked from the edge of the paper to the periphery of the plate in four different directions. The plate was incubated for 16–18 h at 35°C. The presence of growing bacteria within the imipenem antibacterial circle due to carbapenemase production by the test strain was considered as positive.

### PCR amplification of extended-spectrum β-lactamases (ESBL), cephalosporinase (AmpC) and KPC genes and DNA sequence analysis

The primers used for β-lactamases (Table I) were as used in a previous study by Wang *et al*(6). The PCR conditions were: preparation of the template by boiling; denaturation at 94°C for 5 min, then 94°C for 60 sec, 56°C for 30 sec and 72°C for 45 sec for 30 cycles; followed by a final step at 72°C for 10 min.

We used the primers reported by Jiang *et al*(7) for AmpC (Table II). The PCR conditions were: preparation of the template by boiling; denaturation at 94°C for 5 min, then 94°C for 30 sec, 52°C for 30 sec and 72°C for 1 min for 30 cycles; followed by a final step at 72°C for 10 min.

For KPC, in accordance with a previous study (8), we used the following primers: KPC-1 forward, GCTACACCTAGC TCCACCTTC, and reverse ACAGTGGTTGGTAATCCATGC. The PCR conditions were: preparation of the template by boiling; denaturation at 94°C for 5 min, then 94°C for 25 sec, 55°C for 45 sec and 72°C for 60 sec for 35 cycles; followed by a final step at 72°C for 10 min. The purified PCR amplification products were sequenced by Yingwei Jieji (Invitrogen, Shanghai, China) and compared with sequences available in the GenBank database.

### PFGE typing

We used a previously reported method (9) which involved digestion with endonuclease *Xba*I at 37°C for 4 h and electrophoresis at 120°C, 6 V/cm, 5–40 sec for 24 h. The *Xba*I restriction patterns of the genomic DNA of the isolates were analyzed and interpreted according to the criteria of Tenover *et al*(10).

## Results

### Modified Hodge test results

**Figure 1:**
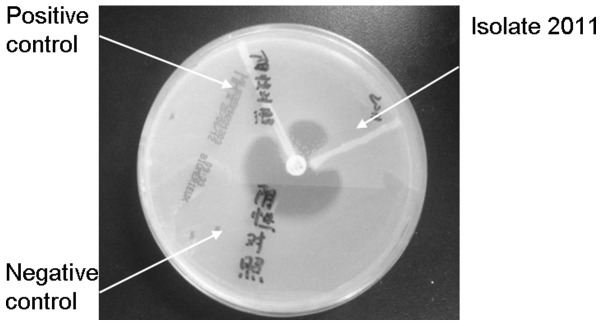
Modified Hodge test result for isolate 2011.

The four imipenem-resistant *Klebsiella pneumoniae* isolates 2011, 2163, 2193 and 2285 revealed an area of inhibition due to carbapenemase production. Fig. 1 presents the result for isolate 2011.

### Analysis of the susceptibility of four Klebsiella pneumoniae isolates to 18 antimicrobial agents

The four isolates were sensitive to polymyxin B and tobramycin, had a moderate degree of resistance to meropenem and a high degree of resistance to the other antibiotics (Table III).

### Screening of extended-spectrum β-lactamases

**Figure 2:**
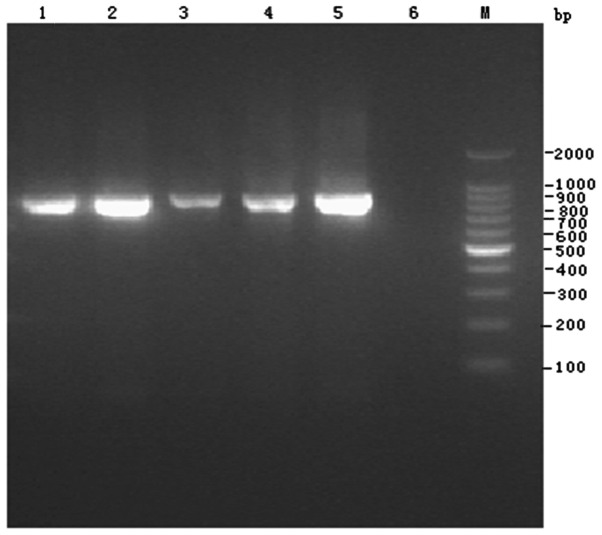
CTX-M-9 β-lactamase PCR results. Lanes 1–4, *Klebsiella pneumoniae* isolates 2011, 2163, 2193 and 2285, respectively; lane 5, positive control; lane 6, negative control; lane 7, marker.

The four isolates all produced extended-spectrum β-lactamases and all had an 857-bp positive band (Fig. 2), which was verified to be CTX-M-9 β-lactamase by sequencing.

### Screening of cephalosporinase

**Figure 3:**
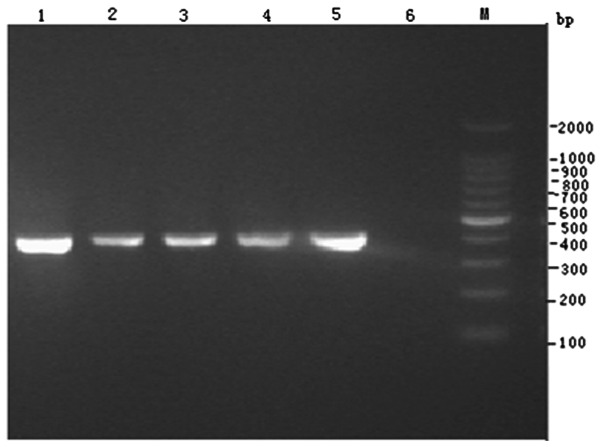
DHA-1 cephalosporinase PCR results. Lanes 1–4, *Klebsiella pneumoniae* isolates 2011, 2163, 2193 and 2285, respectively; lane 5, positive control; lane 6, negative control; lane 7, marker.

The four isolates all produced cephalosporinase and all had a 405-bp positive band (Fig. 3), which was verified to be DHA-1 cephalosporinase by sequencing.

### Screening of carbapenemases

**Figure 4:**
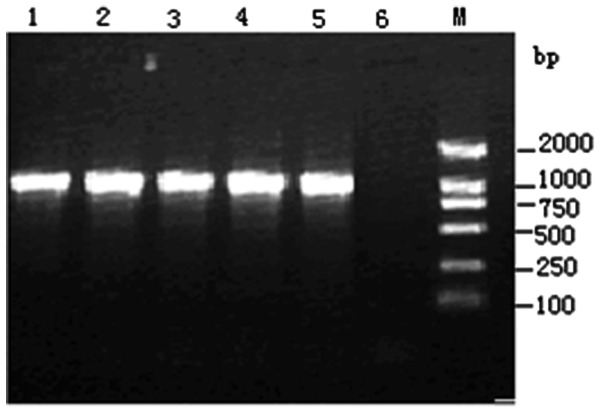
KPC-2 carbapenemase PCR results. Lanes 1–4, *Klebsiella pneumoniae* isolates 2011, 2163, 2193 and 2285; lane 5, positive control; lane 6, negative control; lane 7, marker.

The four isolates all produced carbapenemases and all had a 989-bp positive band (Fig. 4), which was verified to be KPC-2 carbapenemase by sequencing.

### PFGE typing

**Figure 5:**
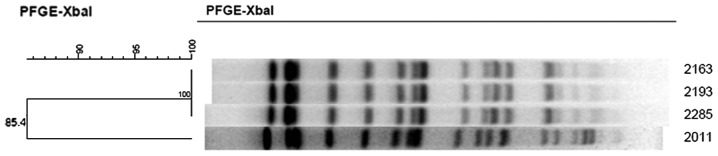
Pulsed-field gel electrophoresis (PFGE) patterns of chromosomal DNA fragments from *Klebsiella pneumoniae* isolates. Lanes 1–4, *Klebsiella pneumoniae* 2011, 2163, 2193 and 2285, respectively.

Fig. 5 presents the PFGE typing of the four isolates. These four isolates were homologous with a difference of 1–3 bands, so they may be considered as four subclones of one pulsed-field-type clone.

## Discussion

Carbapenem antibiotics are highly stable to most β-lactamases, show a high affinity for penicillin-binding proteins and are easily able to enter the periplasmic space by effectively penetrating the bacterial outer membrane. Therefore, they are among the most potent and rapidly acting antibiotics and, in particular, they have unrivalled effects in the treatment of severe infections caused by Enterobacteriaceae. However, reports concerning Enterobacteriaceae which are not sensitive to carbapenem antibiotics have been rapidly increasing in number over the last ten years, the main mechanism of drug resistance being the production of carbapenemases. These enzymes are a class of β-lactamase which are able to significantly hydrolyze imipenem or meropenem. Among the three classes of enzymes A, B and D in the Ambler molecular classification (11), the KPC enzyme is an A type with its gene on a transferable plasmid. It may be horizontally transmitted through plasmids, integrons and gene elements which insert the sequence, and readily leads to an outbreak. However, this plasmid often carries a number of other resistance-determining factors, which is one of the causes of the multidrug resistance of the strains containing it. There are numerous bacteria which produce the KPC enzyme: *Escherichia coli*, *Salmonella*, *Enterobacter cloacae*, acid bacteria isolated from the oak tree (Freund), *Serratia marcescens*, *Proteus*, *Klebsiella oxytoca*, bacteria isolated from the oak tree (Rostock Reber), citric acid bacteria (Freund), *Pseudomonas aeruginosa* and *Pseudomonas putida*, *Acinetobacter spp* and particularly *Klebsiella pneumoniae*. There are 11 subtypes of the KPC enzyme; the KPC subtypes from *Klebsiella pneumoniae* are KPC-1/2, KPC-3, KPC-4, KPC-6, KPC-7, KPC-8 and KPC-11, and the other subtypes have been identified in other bacteria (12). In view of the harmfulness of KPC-producing bacteria, the American Clinical and Laboratory Standards Institute (CLSI) suggested that clinical laboratories should detect the KPC enzyme, test the susceptibility of the *Enterobacter* towards ertapenem, and carry out the adjusted Hodge test for all *Enterobacter* whose MIC values for meropenem and imipenem are greater than 2–4 μg/ml, in order to strengthen the monitoring of the KPC enzyme on a global scale.

In 2008, we identified KPC-2-producing *Klebsiella pneumoniae* in the sputum and blood of a patient who was transferred to our hospital following prolonged therapy in other hospital in Hangzhou. The patient succumbed on the second day after the bacterial infection was detected. Bacteriological specimens were then collected from the ICU environment, medical items, hands of the medical staff and sputum and blood specimens from other patients in the ward. Among the 57 collected specimens, the four isolates 2011, 2163, 2193 and 2285 were shown to be homologous by PFGE. According to the definition of hospital infection outbreaks, which is more than three cases of same type of homologous infection in a medical institution or its patients in a short time, we may conclude that there was a small-scale outbreak of KPC-2-producing *Klebsiella pneumoniae* in our hospital and the source of this medicine-resistant pathogen was another hospital.

The enzyme KPC-2 was detected in all four isolates and was the main cause of their imipenem resistance. In addition, these four isolates also contained the extended-spectrum β-lactamase (ESBL) gene bla_CTX-M-9_ and the cephalosporinase (AmpC) gene bla_DHA-1_, which resulted in multidrug resistance.

Investigation of the epidemic started immediately following the hospital infection outbreak. At the same time, the hospital infection control committee developed infection control programs after investigating the infection source, transmission route and susceptible populations. The detailed measures undertaken were as follows: evacuation of the patients and thorough disinfection of the ICU ward; air disinfection by steaming with 2 g/m^3^ 15% peracetic acid and ozone, mopping the ground and wiping the surfaces of objects with 0.5% peracetic acid; reopening the ICU when the monitoring results were qualified after disinfection; high-pressure steaming or immersion in 0.5% peracetic acid for other medical supplies according to the nature of the materials; and isolation and special care of the infected patient. Following all these measures, no *Klebsiella pneumoniae* with decreased susceptibility to imipenem was identified during tests of air, article surfaces and medical supplies in the ICU ward. Due to the timely detection of the hospital infection outbreak, the control measures effectively prevented the infection from spreading on a larger scale, demonstrating that significance of molecular biology techniques in hospital infection control.

As carbapenem antibiotics are developed and new oral drugs emerge, the quantity of this type of antibiotic used will increase. However, an increased number of drug-resistant strains are likely to arise with an unlimited and extensive use of antibiotics, so this type of efficient and broad-spectrum antibiotic must be used reasonably and prudently. The timely reporting of KPC-producing bacteria in the clinical laboratory is of great significance for guiding the reasonable use of antibiotic, slowing down the emergence of drug-resistant strains and controlling the transmission of and infection with drug-resistant strains.

## Figures and Tables

**Table I:** Primers for β-lactamases.

Target gene	Primer sequence (3′-5′)	Size (bp)
CTX-M	ACGCTTTCCAATGTGCAGTA	
	ACGTCACCAACTGCGCCC	436
CTX-M-1	TTAATTCGTCTCTTCCAGA	
	CAGCGCTTTTGCCGTCTAAG	971
CTX-M-2	CTCAGAGCATTCGCCGCTCA	
	GCGCCGCAGCCAGAATATCC	842
CTX-M-9	GTGACAAAGAGAGTGCAACGG	
	ATGATTCTCCCCCCTGAACCC	857
SHV	GCCTTTATCGGCCCTCACTCAA	
	TTAGCGTTGCCAGTGCTCGATCA	928
TEM	ATAAAATTCTTGAAGACGAAA	
	GACAGTTAGCAATGCTTAATCA	1079

**Table II:** Primers for cephalosporinase (AmpC).

Target genes	Primer	Primer sequence (3′-5′)	Size (bp)
MOX-1, 2; CMY-1, 8, 9, 10, 11	MOXMF	GCTGCTCAAGGAGCACAGGAT	520
	MOXMR	CACATTGACATAGGTGTGGTGC	
LAT-1, 2, 3, 4; BIL-1; CMY-2, 3, 4, 5, 6, 7	CITMF	GGCCAGAACTGACAGGCAAA	462
	CITMR	TTTCTCCTGAACGTGGCTGGC	
DHA-1; DHA-2	DHAMF	AACTTTCACAGGTGTGCTGGGT	405
	DHAMR	CCGTACGCATACTGGCTTTGC	
ACC	ACCMF	AACAGCCTCAGCAGCCGGTTA	346
	ACCMR	TTCGCCGCAATCATCCCTAGC	
MIR-1; ACT-1	EBCMF	TCGGTAAAGCCGATGTTGCGG	302
	EBCMR	CTTCCACTGCGGCTGCCAGTT	
FOX-1, 2, 3, 4, 5	FOXMF	AACATGGGGTATCAGGGAGATG	190
	FOXMR	CAAAGCGCGTAACCGGATTGG	
CMY-4	CMY-4-F	ATGATGAAAAAATCGTTATGC	1100
	CMY-4-R	TTGCAGCTTTTCAAGAATGCGC	

**Table III:** Antimicrobial susceptibility patterns of *Klebsiella pneumoniae*.

A, Minimal inhibitory concentration (MIC)				

	MIC (μg/ml) of isolates			
	
Antimicrobial agents	2163	2193	2285	2011
Imipenem	>16	>16	>16	>16
Ertapenem	>16	>16	>16	>16
Cefoxitin	≥64	≥64	≥64	≥64
Cefepime	≥64	≥64	≥64	≥64
Ciprofloxacin	≥4	≥4	≥4	≥4
Tobramycin	4	4	4	4
Gentamicin	≥16	≥16	≥16	≥16
Aztreonam	≥64	≥64	≥64	≥64
Ceftazidime	≥64	≥64	≥64	≥64
Ceftriaxone	≥64	≥64	≥64	≥64
Piperacillin-tazobactam	≥128	≥128	≥128	≥128
Cefazolin	≥64	≥64	≥64	≥64
Ampicillin	≥32	≥32	≥32	≥32
Ampicillin-sulbactam	≥32	≥32	≥32	≥32

B, Agar disc diffusion (KB testing)				

	KB (mm) of isolates			
	
Antimicrobial agents	2163	2193	2285	2011

Polymyxin B	20	20	21	20
Meropenem	16	16	16	16
Cefoperazone-sulbactam	6	6	6	6
